# Dataset of mechanical properties and electrical conductivity of copper-based alloys

**DOI:** 10.1038/s41597-023-02411-9

**Published:** 2023-07-29

**Authors:** Stéphane Gorsse, Mohamed Gouné, Wei-Chih Lin, Lionel Girard

**Affiliations:** 1 Univ. Bordeaux, CNRS, Bordeaux INP, ICMCB, UMR 5026, 33600 Pessac, France; 2 Department of Materials Science and Engineering, National Tsing Hua University, 101, Sec. 2, Kuang-Fu Road, Hsinchu, 30013 Taiwan, ROC; 3 AMPCO METAL, Route de Chésalles 48, 1723 Marly, Switzerland

**Keywords:** Metals and alloys, Electronic devices

## Abstract

This article presents a collection of data on approximately 150 copper-based alloys. The data compilation is based on articles published since 1993 and consists of about 1830 records. Each record contains a unique set of descriptors, such as composition and processing route, and targets, including properties such as hardness, yield strength, ultimate tensile strength, and electrical conductivity. The dataset includes information on the composition in mass percent of 20 alloying elements, and hundreds of temperature-time thermal treatments and thermomechanical conditions. The database is continually updated and hosted on an open data repository. Some of the data are presented graphically in the article to aid interpretation. This study intends to promote the identification of more sustainable alternatives to Cu-Be alloys, which is particularly relevant in developing non-toxic and environmentally-friendly alloys.

## Background & Summary

Copper is a versatile metal with excellent electrical, thermal, formability, and corrosion-resistant properties, making it a popular choice in a wide range of applications requiring high thermal and electrical conductivity^1–4^. Applications include pumps, tubing, heat exchangers, electrical wires, and contact materials. Copper is also crucial for developing electric cars and energy transition as it is a highly conductive metal that is used in the production of electric motors, wiring, and other components necessary for the efficient transmission and storage of electricity. However, the mechanical strength of pure copper is relatively low at room temperature, making it unsuitable for specific applications that require both strength and conductivity, such as connectors, electromagnetic relays, and aerospace devices. Copper beryllium (Cu-Be) alloys are the most efficient system due to their exceptional combination of high strength, good electrical and thermal conductivity, good wear, and corrosion resistance. However, due to the hazardous nature of beryllium, there is a need for non-toxic and sustainable alternatives. Given this context, finding credible alternatives to Cu-Be alloys is of great importance in developing non-toxic and more sustainable alloys with high strength and high conductivity. This data article presents a compilation of structural and functional properties from published results from 1993 to 2021 for various copper-based alloys and related materials, focusing on alternative systems to Cu-Be alloys. These systems include Cu-Ti and Cu-Ni-Si, which have shown great potential for achieving both high strength and high conductivity. With the increasing demand for non-toxic and sustainable materials, accurate and high-quality data on these alternatives are crucial for further alloy design and development. The data presented in this article can aid researchers and engineers in identifying gaps in the design space, performing data mining, training machine learning models, and developing more sustainable alloys with desirable properties.

## Methods

To locate reliable sources of data on copper alloys, a comprehensive search was conducted on Web of Science and Google Scholar using the keyword “copper-based alloys” in 2022. The search yielded hundreds of potentially useful articles dating from 1993 to 2021, containing experimental data on mechanical properties and conductivity. Bronzes and brasses were excluded as they lie too far from the strength-conductivity Pareto front, as well as Cu-Nb and Cu-Cr composites, to maintain a focus on precipitation-strengthen alloys.

**Fig. 1:**
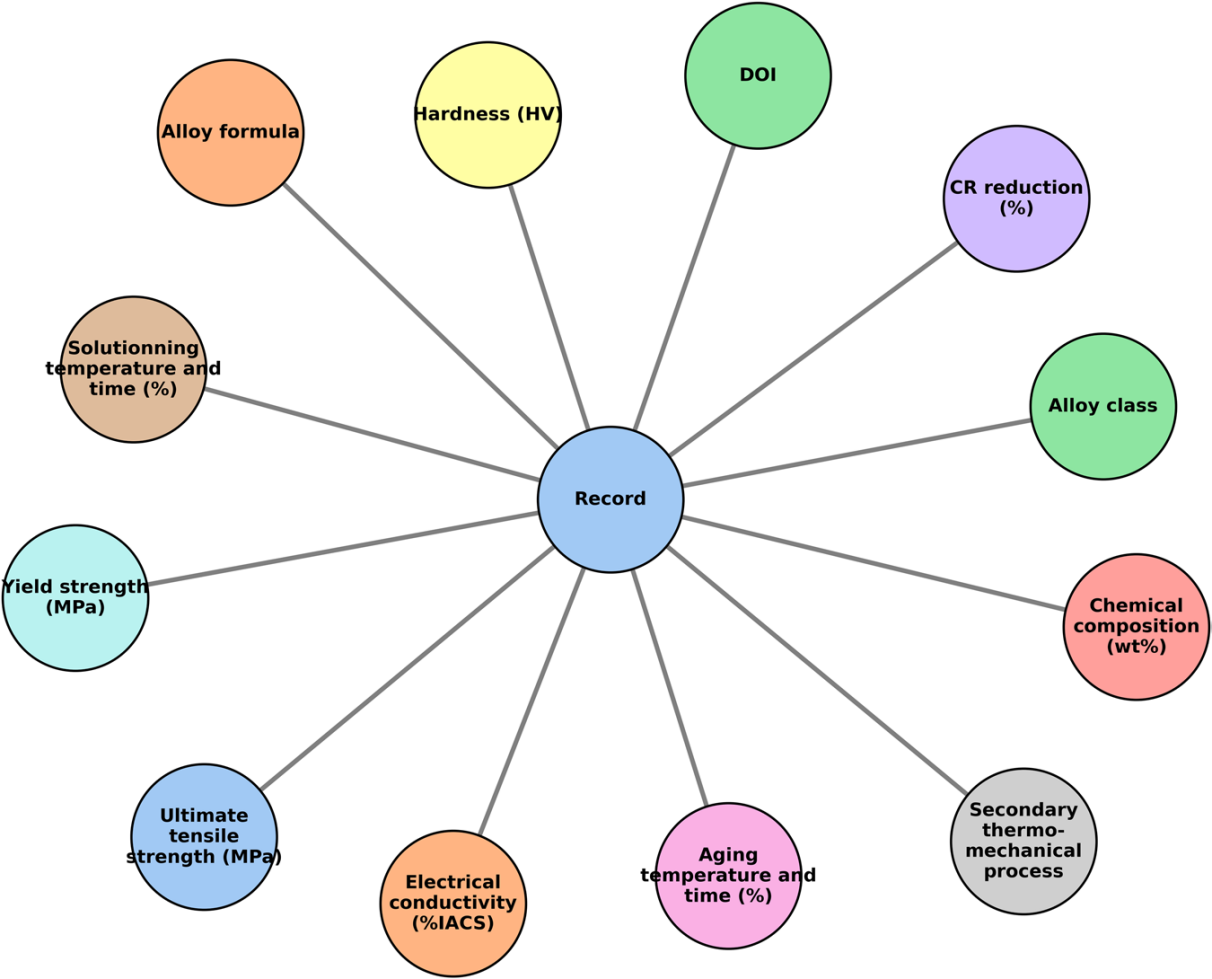
Diagram showing the structure of the dataset. Each line represents a connection. The “Record” element contains all the other elements.

**Fig. 2:**
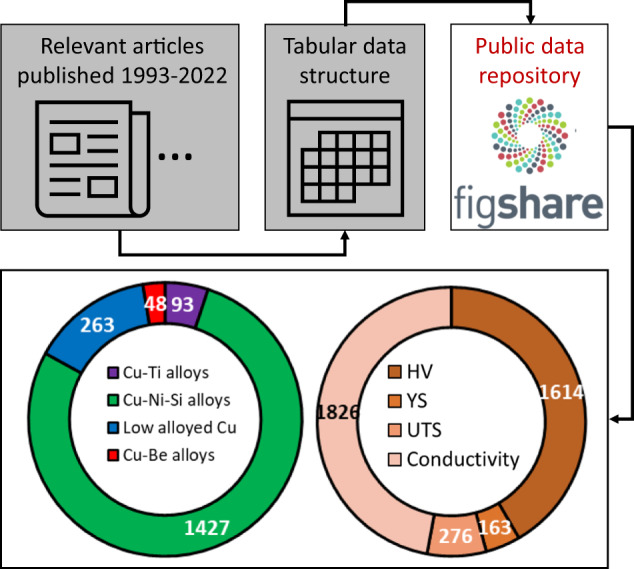
The workflow for constructing the database involves extracting 1831 records from multiple publications and organizing them into a tabular data format. Number of records per alloy classes and properties contained in the resulting database.

The Data Records section describes the process used to extract relevant mechanical property and conductivity data from the articles’ plots, tables, and text. To extract data from plots, we utilized Plotdigitize (https://plotdigitizer.com). Figure 1 illustrates the structure of our dataset. The keywods and structure are documented in the Data Records section. Figure 2 provides an overview of the data extraction workflow.

## Data Records

The complete database on copper-based alloys is available as a csv file via a public online repository at Figshare^5^. The database contains 1831 records from about 50 articles^6–51^. An individual record is defined as having a unique set of descriptors (composition, processing route), targets (properties), and reference combinations.

The data provided in the database are intended to reflect the information available in the literature best. For example, despite the importance of the microstructure on properties, this feature is missing or not properly described from most of the articles, so it is not provided in the database. However, thermal and thermomechanical treatments applied to control the microstructure, which in-fine affect the properties, are recorded, including the temporal dependency of the property targets.

The database records consist of the following fields that are not equally populated for every records due to the lack of literature data:

- Descriptors:
  - Alloy composition: alphabetized nominal alloy composition, in weight percent.
  - Processing method: the conditions under which the alloy was processed after synthesis. They include the temperatures, T_ss_ (K) and T_ag_ (K), and times t_ss_ (h) and t_ag_ (h) of the solid solutionning and precipitation aging thermal treatments, respectively. Cold reduction is filed in when cold rolling was applied prior or after thermal treatment. The secondary thermo-mechanical treatment is indicated by “Y” (yes) or “N” (no) to denote whether additional treatments were applied after aging. These treatments can involve multiple cold rolling and aging processes, among others.
- Targets:
  - Hardness (HV): experimentally reported room temperature Vickers hardness.
  - Yield strength (MPa): measured room temperature yield strength.
  - Ultimate tensile strength (MPa): measured room temperature ultimate tensile strength.
  - Electrical conductivity (%IACS): stands for International Annealed Copper Standard, which is the percentage of room temperature electrical conductivity a material has relative to copper.
- Reference: to ensure the data quality is upheld, a digital object identifier (DOI) link is provided for each record, which links to the original source.

**Fig. 3:**
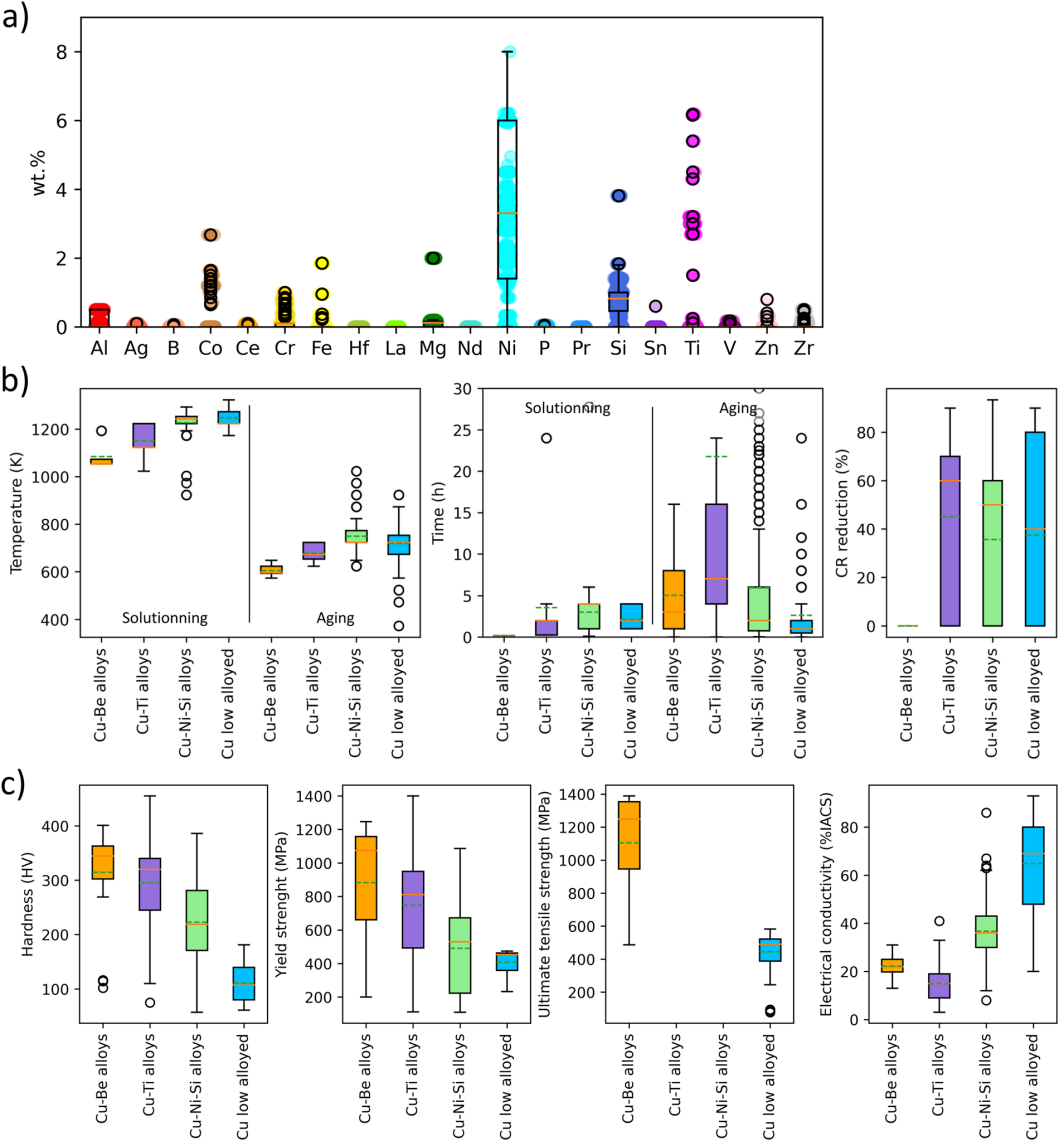
Boxplot showing the statistics for Cu-Be, Cu-Ti, Cu-Ni-Si and low alloyed Cu-based alloys: (**a**) alloying elements, (**b**) temperature and time of the solid solutionning and aging thermal treatments, and cold rolling reduction, (**c**) properties of interest. The box shows the interquartile range between 25th and 75th percentile. The continuous line is the median value, the dashed line shows the average value, the open circles show the outliners, and the whiskers show the minimum and maximum values.

**Fig. 4:**
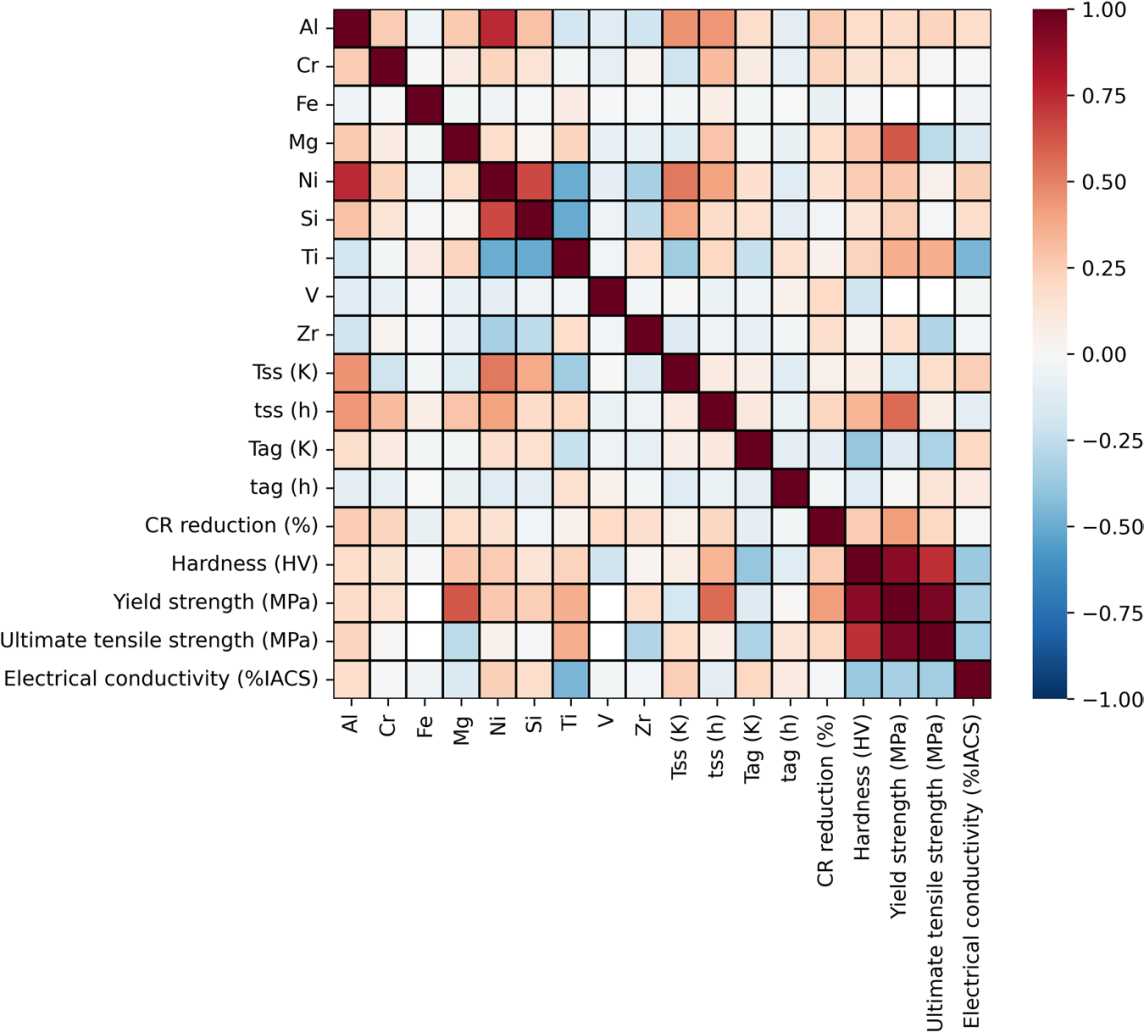
Correlation heatmap showing the strength of the relationship between certain alloying elements, the thermal treatments and the resulting mechanical properties and electrical conductivity element features, for precipitation strengthen alloys (Cu-Be, Cu-Ti and Cu-Ni-Si alloys). It’s noteworthy that there’s a strong negative correlation between the mechanical properties and the electrical conductivity.

Figure 3 provides statistics on the descriptors and property targets extracted and included in the database. In addition to boxplots, Fig. 4 shows a correlation analysis to enhance the understanding of the dataset.

## Technical Validation

The data presented in this article were carefully collected, processed, and thoroughly verified for accuracy by a team of experienced materials scientists and material engineers who possess a deep understanding of metallurgy, copper alloys, and their properties.

The verification process involved a two-step validation procedure. Firstly, during the data extraction phase, we implemented a double-check system. Each data point was independently extracted by two members of our team. Discrepancies between the two extractions were discussed and resolved by referring back to the original source. This process ensured that the data extraction was accurate and consistent. Secondly, we performed a statistical analysis on the collected data. This allowed us to identify any outliers or data points that deviated significantly from the expected values based on the existing literature and theoretical predictions. These data points were re-checked for accuracy.

## Usage Notes

**Fig. 5:**
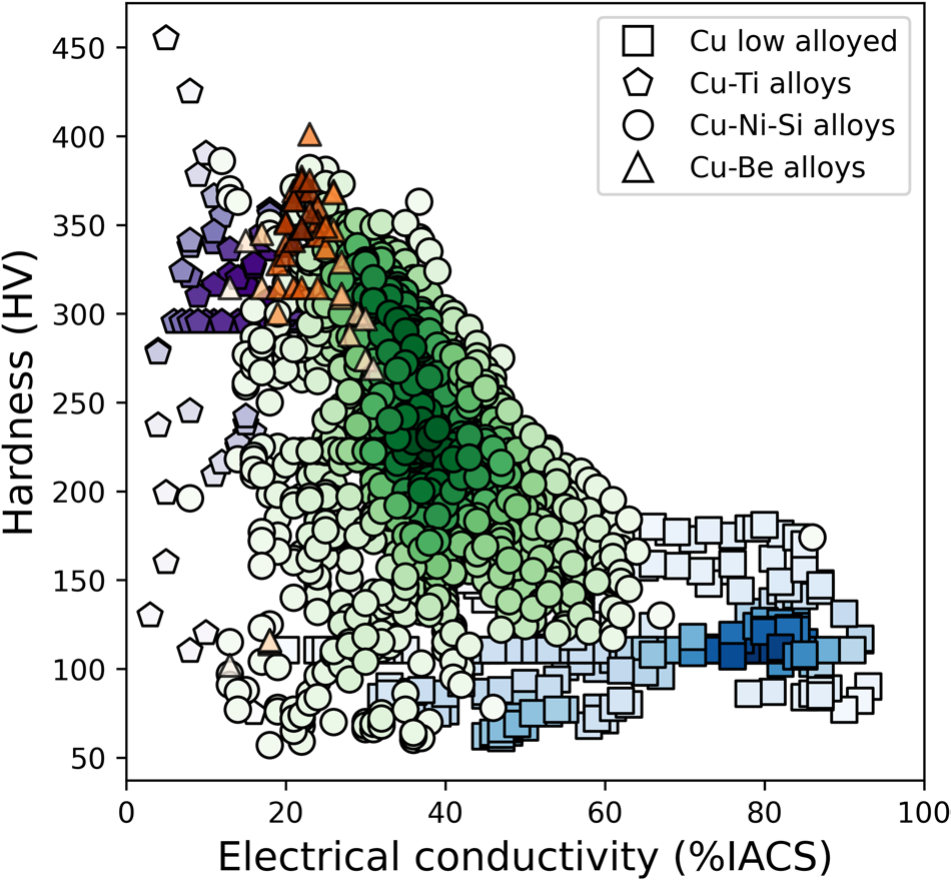
Materials property space for hardness vs electrical conductivity of Cu-based alloys. This chart displays data for about 1830 records for Cu-Be, Cu-Ti, Cu-Ni-Si and low alloyed Cu-based alloys from the current database. The colour predominance of the symbols reflect the alloy class, and the colour gradation shows the local density of the point cloud.

**Fig. 6:**
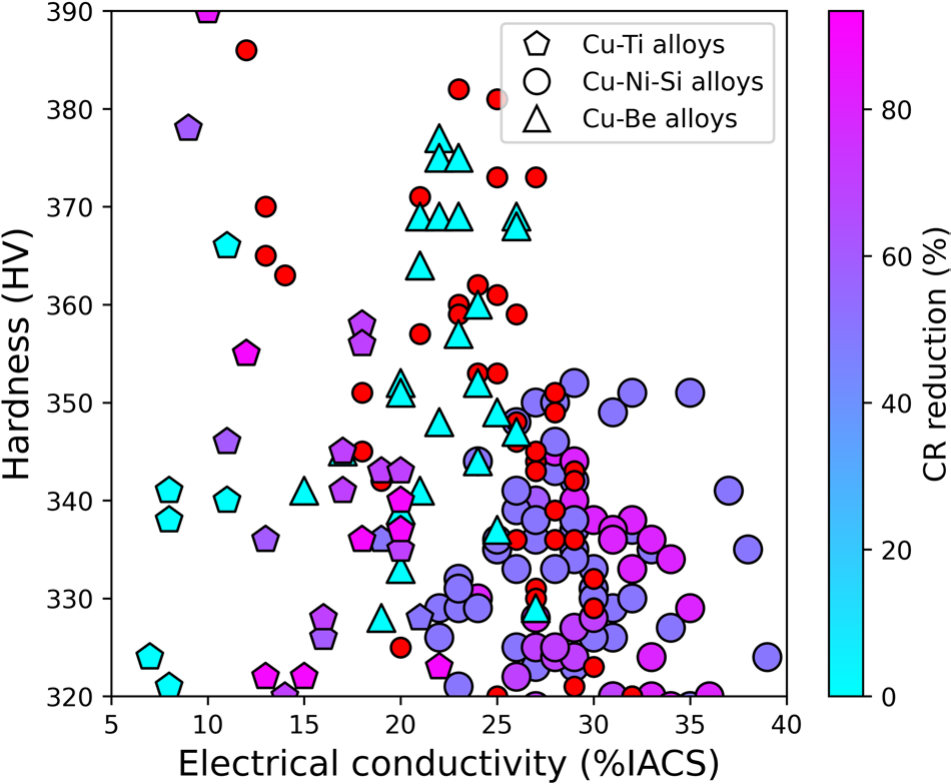
The material property space of Cu-based alloys is examined in terms of hardness (HV) versus electrical conductivity (%IACS), with a specific focus on the region occupied by Cu-Be alloys. The color of the symbols indicates the influence of cold rolling, with red symbols representing the presence of secondary thermo-mechanical treatments.

**Fig. 7:**
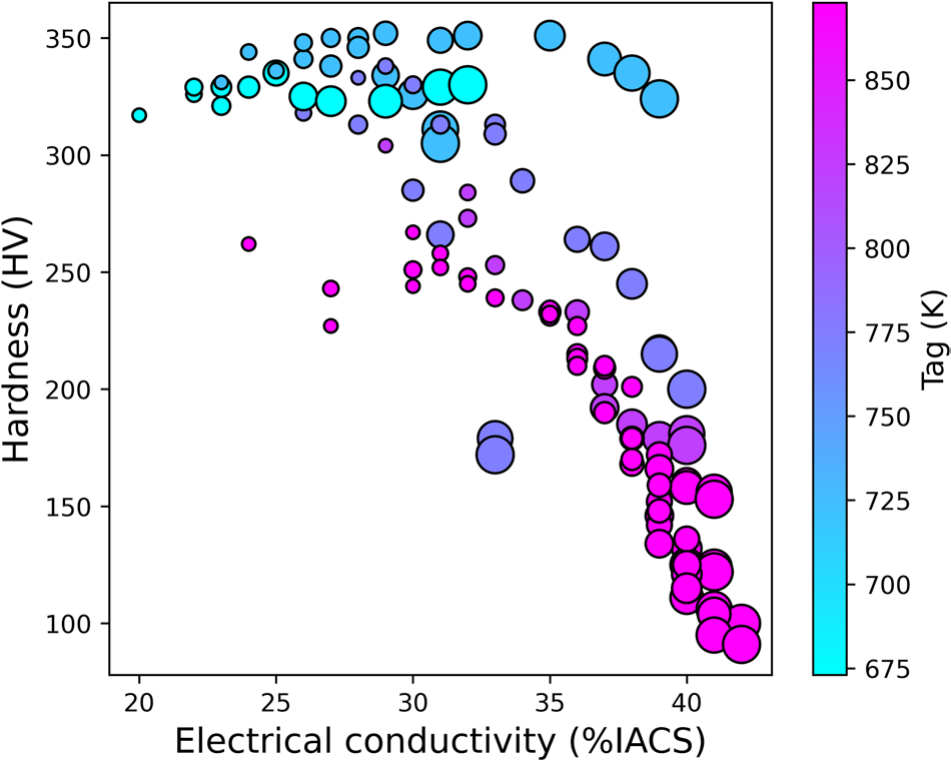
Materials property space for hardness (HV) vs electrical conductivity (%IACS) of the Cu-6Ni-1Si-0.5Al-0.15Mg-0.1Cr alloy (wt.%) in cold rolled condition. Symbol size and color show the effects of aging time and temperature.

This dataset is intended to guide researcher efforts for future Cu-based alloy design and development. It can be helpful as training data for data mining and machine learning applications (e.g. scikit-learn^52^) and produce compelling visualizations in conjunction with standard Python data processing packages (e.g. Matplotlib^53^) as illustrated in Figs. 5–7, where the relationship between hardness and electrical conductivity is displayed. Individual records are shown by symbols with color maps associated with different features. Figure 5 evidences the distinct regions of the property space occupied by different Cu-based alloys classes. The color saturation, corresponding to the density of the data points, highlights the prevalence of low alloyed Cu, Cu-Ni-Si, Cu-Be, and Cu-Ti alloys in specific ranges of (Electrical conductivity, Hardness), namely (80, 120), (30–40, 200–300), (20, 350), and (15, 325) respectively. Figure 6 highlights the region in the upper left corner of the hardness-electrical conductivity space, which is predominantly occupied by Cu-Be alloys. The color map represents the percentage of cold rolling reduction. While a few Cu-Ti and Cu-Ni-Si alloys show potential as competitors to Cu-Be alloys, it is important to note that Cu-Be alloys were not subjected to cold rolling, unlike their counterparts. Additionally, the optimal performance of Cu-Ni-Si alloys overlaps with Cu-Be alloys only when multiple cold rolling and aging treatments are applied. Figure 7 highlights the influence of aging conditions on the properties of a specific alloy composition. It is evident that reducing the aging temperature leads to an increase in hardness, reaching a maximum value at an intermediate aging time. Conversely, the electrical conductivity exhibits an inverse relationship, wherein it increases as both the aging temperature and time increase.

## Data Availability

Data processing, validation and plotting were performed using Excel and Jupyter notebooks^54^ in a Python 3 environment^55^. No custom code has been used.
